# Evaluation of Medicinal Categorization of *Atractylodes japonica* Koidz. by Using Internal Transcribed Spacer Sequencing Analysis and HPLC Fingerprinting Combined with Statistical Tools

**DOI:** 10.1155/2016/2926819

**Published:** 2016-04-12

**Authors:** Jung-Hoon Kim, Eui-Jeong Doh, Guemsan Lee

**Affiliations:** ^1^Division of Pharmacology, School of Korean Medicine, Pusan National University, Yangsan 626-870, Republic of Korea; ^2^Department of Herbology, College of Korean Medicine, Wonkwang University, Iksan 570-749, Republic of Korea; ^3^Center for Metabolic Function Regulation, Wonkwang University, Iksan 570-749, Republic of Korea

## Abstract

*Atractylodes* rhizomes have been used as the herbal medicine “Changchul” or “Baekchul,” according to their clinical purpose, in Korea, China, and Japan. Among the* Atractylodes* species, the medicinal use of* Atractylodes japonica* has been controversial, as it is categorized as both Changchul and Baekchul in those countries, and, moreover, parts of the rhizome have been differently used, depending on age of the plant, in Korea. Chromatographic fingerprinting by using HPLC combined with chemometric analyses and internal transcribed spacer (ITS) sequencing analysis were conducted to classify and identify 34 crude drugs derived from* Atractylodes* rhizomes. The identification of the samples, authenticated by their morphological features as* A. japonica* Koidz. (Changchul and Baekchul),* A. chinensis* Koidz., and* A. macrocephala *Koidz., was confirmed as* A. japonica*,* A. chinensis*, and* A. macrocephala *by ITS sequencing. The results from chemometric analyses showed that the chemical components of the crude drugs from* A. japonica* were significantly different from those from* A. macrocephala* but were similar to those from* A. chinensis*. The analyses also suggested that the categorization by age of* A. japonica* as Changchul or Baekchul is not recommended. The results indicate that* A. japonica* should be categorized as “Changchul” and should not be further categorized by age.

## 1. Introduction

The genus* Atractylodes* (Asteraceae) are perennial herbs distributed in Korea, China, and Japan. Their dried rhizomes have been classified into two kinds of herbal medicines according to their clinical purpose, “Baekchul” (Baizhu in Chinese, Byakujutsu in Japanese) and “Changchul” (Cangzhu in Chinese, Soujutsu in Japanese) [1]. In medicinal applications in Korea, Japan, and China, the rhizomes of* A. lancea* DC. and* A. chinensis* Koidz. have been classified as Changchul, while that of* A. macrocephala* Koidz. has been classified as Baekchul in the pharmacopeias of Korea, China, and Japan [2–4].

However, there has been disagreement between countries in classifying the rhizome of* A. japonica* Koidz.: Korean and Japanese pharmacopeias, as well as some studies from Korea and Japan, have classified the rhizome of* A. japonica* as Baekchul, whereas Chinese studies have classified it as “Gwan-Changchul” (Guan-Cangzhu in Chinese), a type of Changchul, which is not even listed in the Chinese pharmacopeia [5, 6]. Moreover,* A. japonica* is recorded as a synonym of* A. lancea *in the* Flora of China* [7]. Confusion also occurs in local herbal markets in Korea, where the rhizomes of* A. japonica* have been used as Changchul when the fibrous substance has formed after it has grown over two years [8]. The dried rhizomes of* Atractylodes* species can be identified by their morphological features; however, it is difficult to discriminate them by macroscopic observation, a subjective method, due to their morphological similarity. Consequently, the misuse of* Atractylodes* rhizomes may occur when identification is based only on their morphological features. Therefore, strict classification is required for the exact use of* Atractylodes* rhizomes for medicinal purposes.

Genetic identification achieved by hybridization, polymerase chain reaction (PCR), and sequencing techniques is considered more objective, precise, and reliable method for identifying and authenticating herbal species [9, 10]. Internal transcribed spacer (ITS) regions, which are rapidly evolving regions of nuclear ribosomal DNA, have been widely used for the identification of plant materials, and phylogenetic analysis using ITS regions has been conducted to investigate the genetic variability of complicated herbal species [11–13]. It has been reported that the four species of* Atractylodes* (*A. japonica*,* A. macrocephala*,* A. lancea*, and* A. chinensis*) can be distinguished by their different genotypes on ITS sequences [14]. In contrast, one study conducted by PCR-restriction fragment length polymorphism (PCR-RFLP) analysis of the ITS region on nucleotide ribosome DNA (nrDNA) reported that* A. japonica* is not different from* A. macrocephala* [15]. Other studies have demonstrated that the geographical distributions, morphological features, and genetic differentiation between* A. lancea* and* A. chinensis* were not consistent, and* A. japonica* was most closely related to* A. lancea*, according to the results from ITS and* trnL-F* sequences [16, 17]. The results of these studies demonstrate that the classification of* Atractylodes* rhizomes, particularly that of* A. japonica*, remains controversial.

Chemical fingerprinting by using HPLC is an effective and reliable method for the investigation of chemical components in herbal medicines, owing to its high separation efficiency and high detection sensitivity [18]. HPLC fingerprinting also enables a systematic and comprehensive approach to the identification and quantification of the components in herbal medicines [19]. HPLC fingerprinting, combined with chemometric statistical analysis, has been widely used for the quality control of herbal medicines and related herbal products [20, 21]. Moreover, such techniques have also been used to discriminate interspecies differences in herbal medicines, by incorporating principal components analysis (PCA) [22–24]. In previous studies,* A. lancea* was chemically differentiated from* A. chinensis* by HPLC fingerprinting combined with PCA or orthogonal partial least squares-discriminant analysis [25, 26]. However, the chemical discrimination of* Atractylodes* species, using HPLC fingerprinting and chemometric analysis, has not been conducted.

In the present study, chromatographic fingerprinting and chemometric statistical analyses, including PCA, hierarchical clustering analysis (HCA) analysis, and Pearson's correlation coefficient analysis were conducted to classify the crude drugs derived from* Atractylodes* rhizomes. The ITS sequences from nrDNA were also examined to identify the* Atractylodes* species.

## 2. Materials and Methods

### 2.1. Plant Materials and Reagents

Thirty-four samples of crude drugs from* Atractylodes* rhizomes were collected or purchased from the wild, agricultural fields, or local markets in Korea and China. The samples were authenticated by their morphological features through identification criteria by authors. Fifteen rhizomes were identified as* A. chinensis* (coded as “AC”); seven samples of Baekchul and five samples of Changchul were “young” and “aged” dried rhizomes of* A. japonica* (coded as “AJB” and “AJC,” resp.); and seven rhizomes were identified as* A. macrocephala* (coded as “AM”) (Table 1). The voucher specimens have been deposited in the herbarium of the College of Korean Medicine, Woosuk University (Jeonju, Jeonbuk, Korea).

Nine specimens (coded as Arabic number) of* Atractylodes* plants were collected as species reference samples for identification of* Atractylodes* rhizomes in Table 1 and those samples were deposited in the Korea Institute of Oriental Medicine as voucher specimens. The classification result of nine dried-voucher specimens is shown in Table 2.

### 2.2. Preparation of Genomic DNA

The genomic DNA was extracted from the crude drugs of* Atractylodes* rhizomes according to the manuals of NucleoSpin® Plant II kit (Macherey-Nagel, Dueren, Germany). For some samples, 10% cetyl trimethyl ammonium bromide (CTAB) and 0.7 M NaCl were used to remove the phenolic compounds and polysaccharides.

### 2.3. PCR Amplification

For ITS amplification, PCR was performed using T-personal cycler (Biometra, Germany). In brief, 600 nM of primer set of ITS1 (5′-TCCGTAGGTGAACCTGCGG-3′) and ITS4 (5′-TCCTCCGCTTATTGATATGC-3′) [27], 1x BluePreMIX-HF (Macrogen, Korea), and 50 ng of genomic DNA were used for PCR amplification. PCR cycling conditions which were followed by predenaturation process (95°C, 5 min) were as follows: denaturation process (95°C, 30 s); annealing process (52°C, 30 s); extension process (72°C, 40 s) × 36 cycles; and final extension process (72°C, 5 min). The amplified PCR product was separated from other gradients using 1.5% agarose gel electrophoresis after the staining by the addition of Safe-white*™* (abm, Canada). Amplified products were analyzed using MyImage (Seoulin Biotechnology, Korea).

### 2.4. Determination of DNA Sequence of PCR Product

PCR product separated from agarose gel was cloned using MG*™* TOPcloner TA kit (Macrogen, Korea) and DNA sequence of cloned PCR product was determined through the interpretation performed by Macrogen (Korea).

### 2.5. Analysis of DNA Sequence and Preparation of Dendrogram

DNA sequence was analyzed using ClustalW multiple sequence alignment (Bioedit, v7.0.9; available at http://www.mbio.ncsu.edu/BioEdit/page2.html) and the phylogenetic tree was created by using DNADist (Bioedit). To study the relationship of* Atractylodes*, the nucleotide sequences of the genera* Atractylis* and* Carlina* deposited in NCBI GenBank were used. The genera* Brachylaena*,* Cardopatium*,* Cirsium*,* Echinops*,* Phonus*, and* Tarchonanthus* were also used as reference for phylogenetic relationship.* Magnolia heptapeta*,* Zanthoxylum rhoifolium*, and* Lilium cernuum* were used as out-groups in the phylogenetic analyses, based on previous studies [28, 29]. ITS sequences of these taxa were collected from GeneBank in NCBI (the accession numbers were shown in Table 3).

### 2.6. Preparation of Samples for HPLC Analysis

The dried powder of the rhizomes (100 mg) was weighed and then extracted by sonication with 2 mL of ethanol (HPLC grade; Phillipsburg, NJ, USA) for 40 min. The extract was filtered through a 0.45 *μ*m membrane filter (Adventec, Tokyo, Japan) and concentrated in vacuum oven at 45°C. Concentrated extract was dissolved with methanol at the concentration of 5000 *μ*g/mL and filtered through a 0.45 *μ*m membrane filter, prior to HPLC injection.

### 2.7. HPLC Conditions for Chromatographic Fingerprinting

An Agilent 1260 liquid chromatography system (Agilent Technologies, Palo Alto, CA, USA) equipped with an autosampler, degasser, quaternary solvent pump, and diode array detector (DAD) was used for chromatographic fingerprinting. The data were processed by using Chemstation software (Agilent Technologies Inc., USA). The separation of compounds was carried out on a Capcell Pak Mg II C_18_ column (4.6 mm × 250 mm, 5 *μ*m; Shiseido, Tokyo, Japan) with Mg II C_18_ guard cartridge (4.0 mm × 10 mm, S-5; Shiseido, Tokyo, Japan) at 35°C. The flow rate was 1 mL/min and the injection volume was 10 *μ*L. The mobile phase consisted of solvent A (HPLC-grade water; Phillipsburg, NJ, USA) and solvent B (HPLC-grade acetonitrile; Phillipsburg, NJ, USA), with the following gradient elution, 20% (B) over 0–2 min, 20–55% (B) over 2–10 min, 55% (B) over 10–13 min, 55–60% (B) over 13–35 min, 60% (B) over 35–38 min, and 60–75% (B) over 38–50 min, held for 2 min, and then reequilibrated to 20% until the end of the analysis. Detection was performed using a UV detector at the wavelengths of 255, 275, 295, 315, and 340 nm.

### 2.8. Chemometric Statistical Analysis

The 34 samples that were genetically identified and recoded were used for PCA, HCA, and Pearson's correlation analysis. Total 27 peaks were selected as “reference peaks,” and their absolute area was calculated by peak area integration. The 27 reference peaks for the chromatographic fingerprinting, which were representative and >1.0% of total peak area, were chosen at their optimal UV absorption. The absolute area of chosen peak was calculated for chromatographic fingerprinting. A matrix composed of the rows (*Atractylodes* sample) and columns (absolute area of each reference peak) was used for construction of PCA plot, HCA dendrogram, and Pearson's correlation analysis, which were conducted using open-source software R (v.3.1.1).

## 3. Results 

### 3.1. ITS Genotype and Genetic Identification of* Atractylodes *Rhizomes

**Figure 1:**
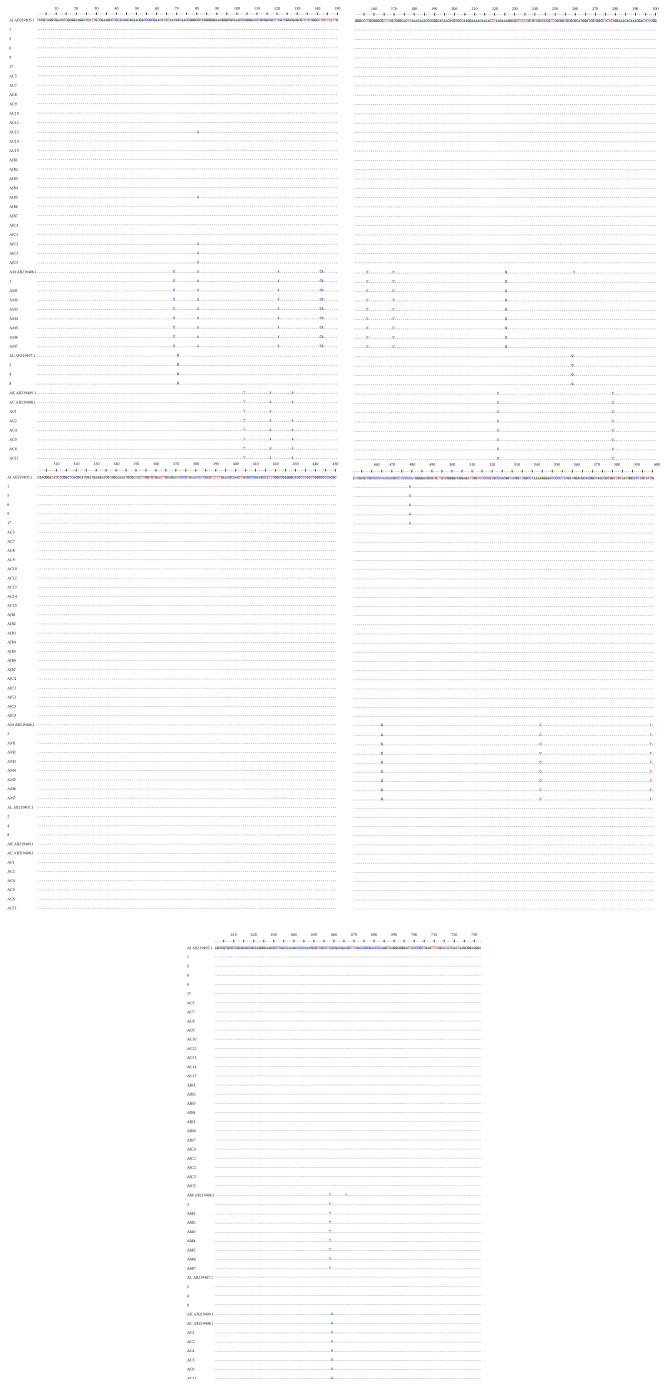
Multiple alignments of ITS nucleotide sequence among the sample listed in Table 1. The dots indicate the consensus nucleotide, and the dashes represent the gaps. AB219405.1 accession numbers of NCBI GenBank for the nucleotide sequences of the ITS for* Atractylodes japonica*; AB219409.1 and AB210407.1 accession numbers of NCBI GenBank for the nucleotide sequences of the ITS for* A. koreana *and* A. lancea*, respectively; AB219406.1 accession number of NCBI GenBank for the nucleotide sequence of ITS for* A. macrocephala*; and AB219408.1 accession number of NCBI GenBank for the nucleotide sequence of ITS for* A. chinensis*. The samples with Arabic number were dried-voucher specimens deposited in the Korea Institute of Oriental Medicine.

The amplification of internal transcribed spacer (ITS) region produced overall 733 bp of nucleotide sequences from 34 samples listed in Table 1 and nine dried-voucher specimens (Figure 1). The determined ITS nucleotide sequence of samples were confirmed by using DNA sequence registered in NCBI GenBank as well as previous paper [14] with comparison of the accession numbers:* A. japonica* (AB219405),* A. macrocephala* (AB219406),* A. lancea* (AB219407),* A. chinensis* (AB219408), and* A. koreana* (AB219409). As presented in Table 4, there was nucleotide substitutions observed on 37 sites on the ITS regions of* Atractylodes* samples. Type 1, ITS sequence of* A. japonica*, showed multiple sequences comparing to other species, while types 2 and 3 were the genotypes of* A. macrocephala* and* A. lancea*, respectively. Type 4, the genotype of* A. chinensis*, was identical to type 5, the genotype of* A. koreana*.

All 7 samples labelled as “AM” were determined as* A. macrocephala* and the difference of DNA sequence between samples was not observed. Among 15 samples labelled as “AC,” AC-1, AC-2, AC-4, AC-5, AC-6, and AC-11 were determined as* A. chinensis*, whereas the rest were* A. japonica*. The sequence of AC-1 was different from that of type 4 by 1 bp (A → G) at nucleotide position, 128 bp, which indicates intraspecific variation. All 7 samples labelled as “AJB” as well as 5 samples labelled as “AJC” were determined as* A. japonica*. AC-13 and AJB-5 were different form that of type 1 by 1 bp (G → A), at nucleotide position, 80 bp. AJC-2, AJC-3, and AJC-5 have 2 different nucleotide positions at 80 bp (G → A) and 481 bp (C → T), showing intraspecific variation.

### 3.2. Genetic Relationship of the* Atractylodes*

**Figure 2:**
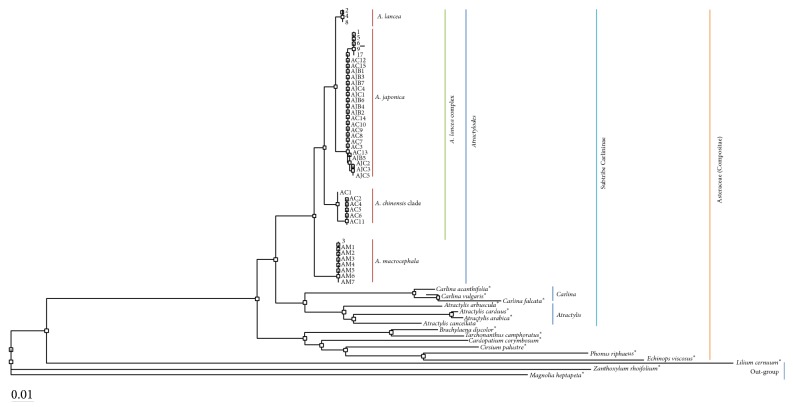
Phylogenic tree from DNADist (Neighbor phylogenetic tree) analysis of the ITS nucleotide sequences. The ITS sequences of taxa with “*∗*” such as* Atactylis*,* Carlina*, and out-group were downloaded from Genbank in NCBI. The samples with Arabic number were dried-voucher specimens deposited in the Korea Institute of Oriental Medicine.

Nine dried-voucher specimens were confirmed as* A. japonica, A. lancea*, and* A. macrocephala*. Thirty-four samples of* Atractylodes* rhizomes were identified as* A. chinensis*,* A. japonica*, and* A. macrocephala*. Phylogenetic classification based on ITS region was constructed and the inferred evolutionary relationships among* Atractylodes* rhizomes were represented on phylogenetic tree. The genus* Atractylodes* is well separated from other close genera and out-groups. The samples of* A. japonica*,* A. lancea*, and* A. chinensis* formed* A. lancea* complex, whereas those of* A. macrocephala* formed their own complex, namely,* A. macrocephala* complex. Five AC samples, AC-2, AC-4, AC-5, AC-6, and AC-11, were involved in* A. chinensis* group. AJB, AJC samples, and the rest AC samples were contained in* A. japonica* group (Figure 2).

Comparing the genotypes listed in Figures 1 and 2 and Table 4, the species of 34 samples in Table 1 of* Atractylodes* rhizomes were recoded as shown in Table 5.

### 3.3. HPLC Fingerprinting of* Atractylodes* Samples

**Figure 3:**
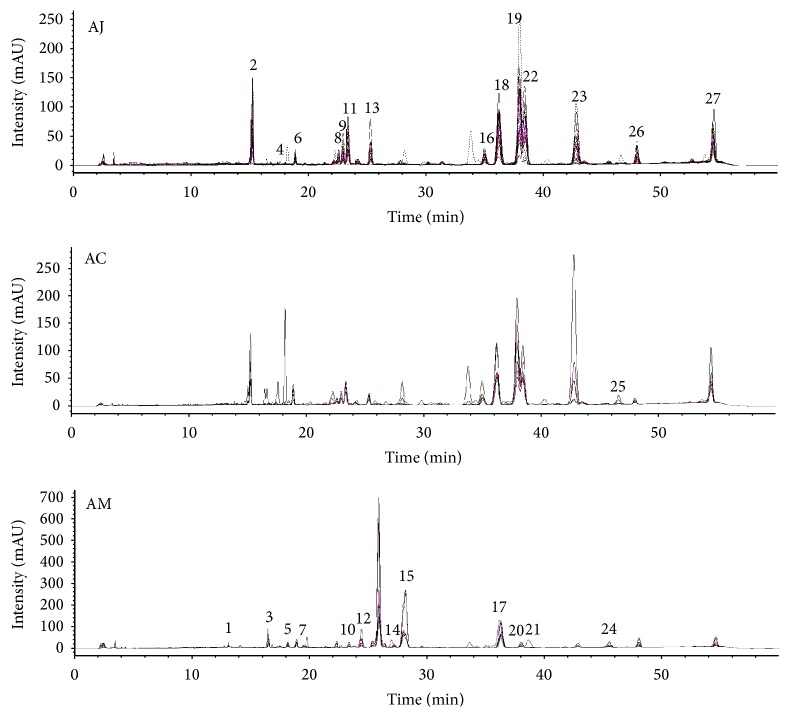
Representative chromatograms of the methanol extracts of AC, AJ, and AM at 255 nm. AC:* A. chinensis* Koidz.; AJ:* A. japonica* Koidz.; AM:* A. macrocephala* Koidz.

**Figure 4:**
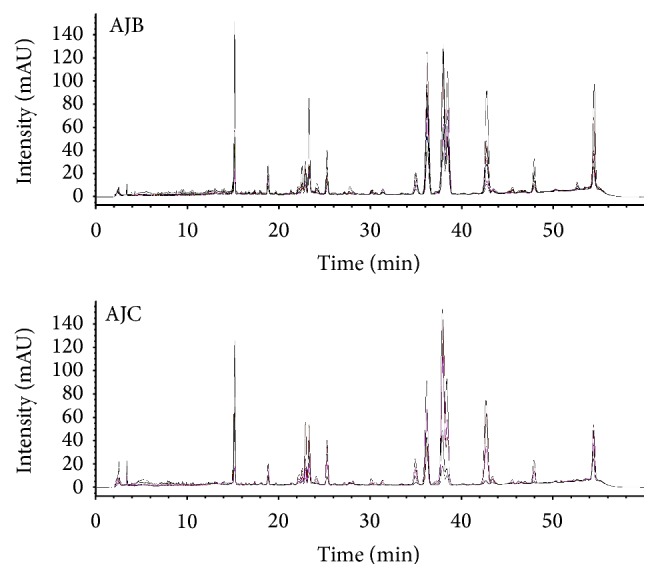
Representative chromatograms of the methanol extracts of AJB and AJC at 255 nm. AJB and AJC:* A. japonica* Koidz.

Through macroscopic observation of the results of HPLC fingerprinting, the chromatograms of* A. japonica* samples (AJBs + AJCs + AJs) and* A. chinensis *samples (ACs) showed peak patterns similar to each other, whereas the chromatograms of* A. macrocephala* samples (AMs) represented different peak patterns compared with those of* A. japonica* and* A. chinensis* samples. Moreover, chromatographic difference between Baekchul samples (AJBs) and Changchul samples (AJCs) was not apparently distinguished (Figures 3 and 4).

### 3.4. Chemometric Statistical Analysis of* Atractylodes* Samples

The results of the chromatographic fingerprinting were further analyzed using principle component analysis (PCA), hierarchical clustering analysis (HCA), and Pearson's correlation analysis to evaluate the correlations between the samples whose code names were redetermined by genetic identification as listed in Table 5.

**Figure 5:**
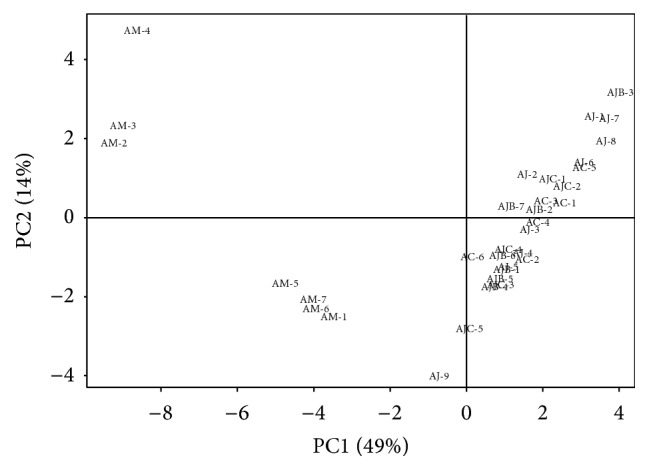
Score plot of principal components (PC1 versus PC2) on the variables (absolute area of reference peaks) with* Atractylodes* samples. PC1 and PC2 represent 49% and 14% of the total variance, respectively. AC:* A. chinensis *Koidz.; AJ, AJB, and AJC:* A. japonica* Koidz.; AM:* A. macrocephala* Koidz.

Principle component 1 (PC1) score divided the samples of “*A. japonica* +* A. chinensis*” group in the positive plot with exception of AC-6, AJ-9, and AJC-5, while “*A. macrocephala*” group was in the negative plot. PC2 score further differentiated the samples having positive PC1 scores into positive PC2 plot (AC-1, AC-3, AC-5, AJ-1, AJ-2, AJ-6, AJ-7, AJ-8, AJB-2, AJB-3, AJB-7, AJC-1, and AJC-2) and negative PC2 plot (AC-2, AC-4, AJ-3, AJ-4, AJ-5, AJB-1, AJB-4, AJB-5, AJB-6, AJC-3, and AJC-4) and samples having negative PC1 scores into positive PC2 (AM-1, AM-5, AM-6, and AM-7) and negative PC2 (AM-2, AM-3, and AM-4) (Figure 5).

**Figure 6:**
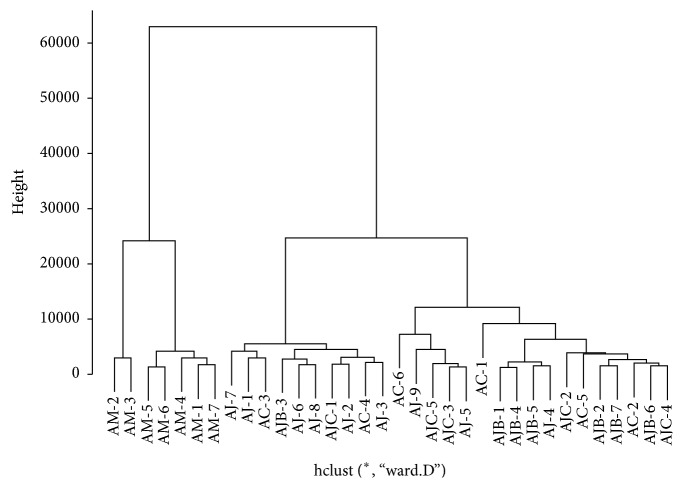
Hierarchical clustering analysis of* Atractylodes* samples. AC:* A. chinensis* Koidz.; AJ, AJB, and AJC:* A. japonica* Koidz.; AM:* A. macrocephala* Koidz.

HCA results also showed similar result obtained from PCA. Below the height of 30000, while the samples of* A. macrocephala* formed their own clustering, those of* A. japonica* and* A. chinensis* were gathered together, where* A. japonica* samples and* A. chinensis *samples were mixed. Therefore, apparent discrimination between* A. japonica* samples and* A. chinensis* samples was not observed. Moreover, as seen in PCA score plot, AJB and AJC samples were not clearly distinguished in HCA dendrogram, as they were grouped undistinguishably within the same levels (Figure 6).

**Figure 7:**
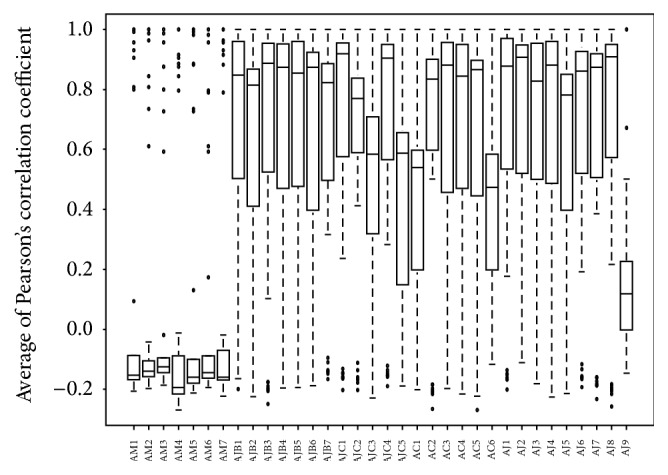
Average coefficients of Pearson's correlation of 34* Atractylodes* samples. AC:* A. chinensis* Koidz.; AJ, AJB, and AJC:* A. japonica* Koidz.; AM:* A. macrocephala* Koidz.

Box plot of average Pearson's correlation coefficient (*r*) showed two distinct sample groups, the group of* A. macrocephala* samples and the group of* A. japonica* and* A. chinensis* samples. The average coefficients of* A. macrocephala* samples ranged from −0.2 to 0.0, whereas those of* A. japonica* and* A. chinensis* samples ranged from 0.5 to 1.0, except for AJ-9 showing the average coefficient between 0.0 and 0.2. Furthermore, AJB and AJC samples showed similar values of average Pearson's coefficients (Figure 7).

## 4. Discussion

Discrimination of herbal medicines according to their therapeutic effect is most important in using herbal medicines as therapeutic agents. To achieve such purpose, correct classification of herbal medicines should be preceded at their species levels. The rhizome of* A. japonica* has been at the center of controversy as it is differently classified in many countries; Korea and Japan have used that as “Baekchul,” whereas China has used that as “Changchul.” Furthermore, the rhizome of* A. japonica* is further divided into “Baekchul” and “Changchul” by the age; tuberous part grown for less than a year is used as Baekchul, while fibrous part grown for more than two years is used as Changchul. Hence, we classified* Atractylodes* samples which were genetically identified by ITS genotypes using analytical tools combined with chemometric statistics, in order to propose correct categorization of* Atractylodes* rhizomes, especially* A. japonica* rhizomes.

### 4.1. Genetic Identification of* Atractylodes* Samples

*Atractylodes*,* Atractylis,* and* Carlina* belong to subtribe Carlininae [28]. ITS sequence of* A. japonica* showing multiple sequences indicates that there are diverse intraspecific variations in* A. japonica* samples. The genotype of* A. chinensis* was identical to that of* A. koreana*. As the distribution of* A. koreana* is limited in Shandong and Liaoning area,* A. koreana* has been possibly derived from* A. chinensis* [14, 17]. The samples of* A. japonica*,* A. lancea*, and* A. chinensis* formed* A. lancea* complex, in accordance with the previous research [17].* A. japonica* is most closely related to* A. lancea* and next related to* A. chinensis*, as suggested in previous researches [17, 30, 31]. The samples identified as* A. japonica* were obviously separated from those identified as* A. macrocephala* by DNA sequencing analysis [14, 16, 32]. According to the results, there was difference in classification between morphological identification and genetic identification which might be caused by morphological similarity.

### 4.2. Classification of* Atractylodes* Samples Using Chemometric Analysis

Principal components analysis (PCA) was performed for the clustering of the samples and for investigating the relationships among the samples using principal components on the PCA plot. Principal component 1 (PC1) explains most of the variance, and PC2, which is orthogonal to PC1, represents most of the variance not explained by PC1 [33]. HCA is another clustering method which classifies similar objects mathematically into the same group and the classifications were represented as tree diagram called dendrogram [34]. Pearson's correlation coefficient of each sample was calculated to evaluate the correlations between samples (−1 ≤* r* ≤ +1; the closer the *r* is to 1, the more correlated the two fingerprints are) [35].

From the results of PCA, HCA, and Pearson's correlation analysis, the* A. japonica* samples were obviously distinct from* A. macrocephala *samples but distributed close to the* A. chinensis* samples without apparent separation of AJBs and AJCs samples. These results indicate that* A. japonica* is chemically different from* A. macrocephala*; however, the crude drugs derived from* A. japonica* and* A. chinensis* were not clearly distinguishable by their chemical components. These results indicate that the therapeutic effects of* A. japonica* and* A. chinensis* whose effects can be exerted by the chemical components are thought to be presumably analogous [36], although they are genetically different. Moreover, the separation of* A. japonica* rhizomes as “Baekchul” and “Changchul” is not recommended because they had the closest PC scores, equal levels of hierarchies, and similar Pearson's coefficients [37]; therefore, AJBs and AJCs can be assumed to be the same medicinal parts.

### 4.3. Assured Medicinal Categorization of* Atractylodes japonica *Rhizomes

The categorization of* A. japonica* as Baekchul in Korea and Japan, therefore, should be removed, because its relationship to* A. macrocephala*, the only species regarded as Baekchul in China, is not close, which supports the results from previous studies [14, 17, 32, 38]. Moreover, we do not recommend the segregation of* A. japonica* into Baekchul and Changchul, because the segregated crude drugs of* A. japonica* are not distinguishable by their chemical and genetic characteristics. This suggests that there is the lack of chemical or genetic reason to categorize the rhizome of* A. japonica* as Changchul and Baekchul based on plant age.

Therefore, we propose that* A. japonica* should be separated from* A. macrocephala* and should be used as “Changchul” as the Chinese literatures define it, regardless of its age. Further biological and clinical experiments are necessary to confirm the results of the present study.

In conclusion, the 34 crude drugs derived from* Atractylodes* rhizomes were identified using ITS sequencing analysis and classified using HPLC fingerprinting and statistical tools such as PCA, HCA, and Pearson's correlation analysis. The macroscopically authenticated* Atractylodes* samples were genetically identified as* A. japonica*,* A. chinensis*, and* A. macrocephala* by ITS DNA sequencing. The hyphenated HPLC fingerprinting and statistical analyses showed that the chemical components of* A. japonica* were not related to those of* A. macrocephala* but related to those of* A. chinensis*. Moreover, the rhizome of* A. japonica* could not be segregated as Baekchul and Changchul by its age. The results from these chemical and genetic analyses demonstrate that* A. japonica* should be classified as “Changchul” and should not be classified by plant age.

## Figures and Tables

**Table 1:** Morphological identification of the crude drugs of *Atractylodes* rhizomes.

Code	Species	Origin	Code	Species	Origin
AC-1	*A. chinensis *Koidz.	China	AJB-3	*A. japonica* Koidz.	China
AC-2	*A. chinensis *Koidz.	China	AJB-4	*A. japonica* Koidz.	Unclear
AC-3	*A. chinensis *Koidz.	China	AJB-5	*A. japonica* Koidz.	Unclear
AC-4	*A. chinensis *Koidz.	China	AJB-6	*A. japonica* Koidz.	Unclear
AC-5	*A. chinensis *Koidz.	China	AJB-7	*A. japonica* Koidz.	Korea
AC-6	*A. chinensis *Koidz.	Korea	AJC-1	*A. japonica* Koidz.	Korea
AC-7	*A. chinensis *Koidz.	Korea	AJC-2	*A. japonica* Koidz.	Unclear
AC-8	*A. chinensis *Koidz.	China	AJC-3	*A. japonica* Koidz.	Unclear
AC-9	*A. chinensis *Koidz.	China	AJC-4	*A. japonica* Koidz.	Korea
AC-10	*A. chinensis *Koidz.	China	AJC-5	*A. japonica* Koidz.	Unclear
AC-11	*A. chinensis *Koidz.	China	AM-1	*A. macrocephala* Koidz.	China
AC-12	*A. chinensis *Koidz.	China	AM-2	*A. macrocephala* Koidz.	China
AC-13	*A. chinensis *Koidz.	China	AM-3	*A. macrocephala* Koidz.	China
AC-14	*A. chinensis *Koidz.	China	AM-4	*A. macrocephala* Koidz.	China
AC-15	*A. chinensis *Koidz.	China	AM-5	*A. macrocephala* Koidz.	China
AJB-1	*A. japonica* Koidz.	Korea	AM-6	*A. macrocephala* Koidz.	China
AJB-2	*A. japonica* Koidz.	Korea	AM-7	*A. macrocephala* Koidz.	China

**Table 2:** Identification of dried-voucher specimens coded as Arabic numbers.

Number	Identification
1	*A. japonica* Koidzumi
2	*A. lancea* de Candolle
3	*A. lancea* de Candolle
4	*A. macrocephala* Koidzumi
5	*A. japonica* Koidzumi
6	*A. japonica* Koidzumi
8	*A. lancea* de Candolle
9	*A. japonica* Koidzumi
17	*A. japonica* Koidzumi

**Table 3:** The GenBank accession number of species that are used in phylogenetic tree analysis.

Species	GenBank accession number
*Atractylis cancellata*	AY826231.1
*Atractylis arabica*	KF850563.1
*Atractylis carduus*	AY826232.1
*Atractylis arbuscula*	KF301215.1
*Carlina falcata*	AY826243.1
*Carlina vulgaris*	KF301217.1
*Carlina acanthifolia*	KF301216.1
*Echinops viscosus*	AY826283.1
*Phonus riphaeus*	AY826310.1
*Cirsium palustre*	EU143268.1
*Cardopatium corymbosum*	AY826238.1
*Tarchonanthus camphoratus*	AY826340.1
*Brachylaena discolor*	AY826236.1
*Magnolia heptapeta*	AY858638.1
*Zanthoxylum rhoifolium*	KC502933.1
*Lilium cernuum*	HQ686064.1

**Table 4:** Genotypes of ITS nucleotide sequences deposited in the NCBI GenBank for recognition in the *Atractylodes* species.

Nucleotide position	69	71	81	104	117	120	121	128	134	135	141	142	143	145	149	157	170	177	222	226	259	260	279	465	481	494	502	529	530	534	544	560	572	599	658	659	666
Genotype																																					
Type 1	T	A	G	C	G	**Y**	C	G	**M**	**Y**	**R**	T	C	Y	Y	T	T	R	A	A	C	G	T	**W**	**Y**	**Y**	**R**	**Y**	**R**	**Y**	A	**Y**	**W**	C	C	G	C
Type 2	C	A	A	C	G	C	A	G	C	C	G	C	A	C	C	C	C	G	A	G	C	**R**	T	G	C	C	G	C	G	C	C	C	T	T	T	G	**Y**
Type 3	T	G	G	C	G	C	C	G	C	C	G	T	C	C	C	T	T	A	A	A	G	G	T	A	C	C	G	T	G	C	A	C	A	C	C	G	C
Type 4	T	A	G	T	A	C	C	A	C	C	G	T	C	C	T	T	T	G	C	A	C	G	C	T	C	C	G	C	G	C	A	C	T	C	C	A	C
Type 5	T	A	G	T	A	C	C	A	C	C	G	T	C	C	T	T	T	G	C	A	C	G	C	T	C	C	G	C	G	C	A	C	T	C	C	A	C

^*∗*^Bold characters indicate nucleotide additives, M = A and C; R = A and G; W = A and T; Y = C and T. Type 1 genotype resulted from deposited ITS nucleotide sequences in NCBI Genbank of *Atractylodes japonica*. Type 2 and type 3 represent *A. macrocephala* and *A. lancea*. Type 4 and type 5 represent *A. chinensis* and *A. koreana*.

**Table 5:** Genetic identification of original species of *Atractylodes* rhizomes listed in Table 1.

Code	Genetically identified species	Recode	Code	Genetically identified species	Recode
AC-1	*A. chinensis*	AC-1	AJB-3	*A. japonica*	AJB-3
AC-2	*A. chinensis*	AC-2	AJB-4	*A. japonica*	AJB-4
AC-3	*A. japonica*	AJ-1	AJB-5	*A. japonica*	AJB-5
AC-4	*A. chinensis*	AC-3	AJB-6	*A. japonica*	AJB-6
AC-5	*A. chinensis*	AC-4	AJB-7	*A. japonica*	AJB-7
AC-6	*A. chinensis*	AC-5	AJC-1	*A. japonica*	AJC-1
AC-7	*A. japonica*	AJ-2	AJC-2	*A. japonica*	AJC-2
AC-8	*A. japonica*	AJ-3	AJC-3	*A. japonica*	AJC-3
AC-9	*A. japonica*	AJ-4	AJC-4	*A. japonica*	AJC-4
AC-10	*A. japonica*	AJ-5	AJC-5	*A. japonica*	AJC-5
AC-11	*A. chinensis*	AC-6	AM-1	*A. macrocephala*	AM-1
AC-12	*A. japonica*	AJ-6	AM-2	*A. macrocephala*	AM-2
AC-13	*A. japonica*	AJ-7	AM-3	*A. macrocephala*	AM-3
AC-14	*A. japonica*	AJ-8	AM-4	*A. macrocephala*	AM-4
AC-15	*A. japonica*	AJ-9	AM-5	*A. macrocephala*	AM-5
AJB-1	*A. japonica*	AJB-1	AM-6	*A. macrocephala*	AM-6
AJB-2	*A. japonica*	AJB-2	AM-7	*A. macrocephala*	AM-7
